# Wastewater from the Arenga Starch Industry as a Potential Medium for Bacterial Cellulose and Cellulose Acetate Production

**DOI:** 10.3390/polym15040870

**Published:** 2023-02-09

**Authors:** Rahmayetty, Fatah Sulaiman

**Affiliations:** 1 Chemical Engineering Department, Universitas Sultan Ageng Tirtayasa, Jl. Jendral Sudirman Km 3, Cilegon 42435, Banten, Indonesia; 2 Center of Excellence Local Food Innovation, Universitas Sultan Ageng Tirtayasa, Jl. Raya Palka Km 3, Sindangsari, Kabupaten Serang 42118, Banten, Indonesia; 3 Applied Biomaterial and Product Engineering Laboratory, Center of Excellence, Universitas Sultan Ageng Tirtayasa, Jl. Jendral Sudirman Km 3, Cilegon 42435, Banten, Indonesia

**Keywords:** bacterial cellulose, cellulose acetate, wastewater of the Arenga starch industry

## Abstract

Wastewater from the Arenga starch industry (WWAS) contains a high chemical oxygen demand (COD) concentration, so it has to be treated before being discharged into water bodies. Therefore, the purpose of this study was to utilize WWAS as a medium for bacterial cellulose (BC) and cellulose acetate (CA) production. This study consisted of the production of BC through fermentation and the production of CA through acetylation. Fermentation was conducted under static batch conditions with various initial pHs and sucrose additions, while acetylation was conducted with various BC–acetic anhydride ratios. The results of this study showed that the maximum BC production of 505.6 g/L of the culture medium was obtained under the optimal conditions of a sucrose addition of 200 g/L, an initial medium pH of 4.5, and a cultivation time of 14 d. Furthermore, a BC–acetic anhydride ratio of 1:3 resulted in CA being suitable as a biofilm raw material with a yield of 81.49%, an acetyl content of 39.82%, a degree of substitution of 2.456, and a degree of crystallinity of 36.7%. FT−IR, ^1^H and ^13^C NMR, XRD, and SEM analyses confirmed the successful process of acetylation of BC to CA.

## 1. Introduction

Sugar palm (*Arenga pinnata*) is a tall and large palm with a single unbranched stem that is part of the Palmae family and is naturally a forest species [1,2]. *Arenga pinnata* is dominantly found in tropical areas, such as Indonesia [2]. In Indonesia, the total areas of *Arenga pinnata* plantations are estimated to reach 61,924 ha across 26 provinces [3]. One product obtained from the processing of *Arenga pinnata* is Arenga starch, which is extracted from the trunks of *Arenga pinnata* trees [1,4]. Processing 10 tons of raw materials can produce 2–3 tons of Arenga starch and 10 m^3^ of wastewater [5]. This wastewater is known as the wastewater of Arenga starch (WWAS). Aside from its high volume, WWAS contains high chemical oxygen demand (COD) and biological oxygen demand (BOD) concentrations, which have been reported as 8740 and 2985 mg/L, respectively [5], so it cannot be discharged directly into water bodies because it has a high potential to cause water pollution.

WWAS contains large amounts of organic matter and ammonia, has a high carbon-to-nitrogen (C/N) ratio of 15:1 [6], has high concentrations of metal ions of Mn, Zn, and Cu of 0.11, 0.305, and 0.121 mg/L, respectively [4], and has a low degree of acidity, with pH values in the range of 3.8–5.2 [7]. Carbon and nitrogen are macronutrients and the metal ions are micronutrients for bacterial growth [8]. Therefore, WWAS has the potential to be utilized as a medium for bacterial cellulose (BC) production. Based on our study of the literature, the utilization of WWAS as a medium for BC production has not yet been reported in previous studies. On the other hand, the potency of some other liquid wastes as a medium for BC production was studied and reported by previous authors; these wastes were distillery effluents [9,10], tobacco waste extracts [11], molasses [12,13,14,15,16], whey [17], wastewater of candied jujube [18], stillage wastewater [19], olive oil mill wastewater [20], and glycerol [21].

Bacterial cellulose (BC) is an extracellular polysaccharide that results from the activities of some bacterial species that belong to the genera *Rhizobium*, *Acetobacter*, *Agrobacterium*, *Achromobacter*, *Aerobacter*, *Azotobacter*, *Gluconacetobacter*, *Salmonella*, *Escherichia*, *Pseudomonas*, and *Sarcina* [22,23,24,25]. Among the *Acetobacter* strains, *Acetobacter xylinum* is the most efficient genus in producing BC when using a liquid culture [26,27]. The genus *Acetobacter* is used the most often in the production of BC, due to its ability to metabolize a wide range of carbon/nitrogen sources [28,29]. Bacterial activities when producing BC are mainly affected by the initial medium pH and the carbon sources used [30]. For example, fermentation that used distillery effluents as a medium with a sucrose addition resulted in a BC yield of 8.11 g/L at a pH of 4.75 [10]; fermentation that used apple juice waste as a medium with a sucrose addition resulted in a BC yield of 64 g/L at a pH of 5.5 [31]; fermentation that used coconut water as a medium with a sucrose addition resulted in a BC yield value in the range of 60–100 g/L in wet weight at a pH of 4.5 [32]; fermentation that used the wastewater of candied jujube as a medium with an ammonium citrate addition resulted in a BC yield of 2.25 g/L in dry weight at a pH of 6 [18]; fermentation that used pineapple peel juice as a medium with glucose, fructose, and sucrose additions resulted in a BC yield of 2.8 g/L at a pH of 3.5 [33], and fermentation that used stillage wastewater as a medium with a glucose addition resulted in a BC yield of 3.05 g/L at a pH of 6 [19].

One of the most interesting products of BC is cellulose acetate (CA). CA has been widely used in many fields, including textiles, coatings, films, filters, and synthetic polymeric membranes [34]. CA has excellent properties, such as its biodegradability, hydrophilic qualities, ease of processing, non-toxicity, and renewability [35]. Therefore, CA has great potential for use as a raw material for biofilms. The conversion of BC into CA is carried out through an acetylation process with sulfuric acid, acetic anhydride, and acetic acid as a catalyst, an acetylating agent, and a solvent, respectively [36,37,38], which is followed by a hydrolysis process. The quality of CA, including its characteristics and solubility in solvents, is determined by the degree of substitution (DS) and the acetyl content [39]. In the application of CA as a biofilm, the acetyl content in CA must be in the range of 36.5–42.2% to obtain a biofilm with good physical and mechanical characteristics [40]. The acetyl cellulose content is largely determined by the ratio between cellulose and acetic anhydride (AcAn).

Based on the description provided above, we hypothesized that WWAS, which is abundant and contains large amounts of organic compounds, nitrogen, and metal ions, can be utilized as a medium for producing BC, which can then be used to produce CA. This study is unique because there are no previous studies that have examined the potency of WWAS as a medium for BC and CA production. The significance of this study is that inexpensive and environmentally friendly BC and CA can be industrially produced on a large scale in Indonesia in the future. The purpose of this study was to synthesize and characterize BC and CA products by using WWAS as a medium. In the synthesis of BC, the effects of the initial medium pH and sucrose additions to the WWAS medium on the yield of BC were investigated. Furthermore, in the synthesis of CA, the effects of the BC–acetic anhydride ratio (BC:AcAn) in the acetylation process on the yield and characteristics of CA were investigated. The chemical, structural, and microstructural characteristics of the BC and CA products were studied to determine the promise of the products for application as inexpensive and environmentally friendly biomaterials.

## 2. Materials and Methods

### 2.1. Materials

The strain *Acetobacter xylinum* that was used to produce BC was purchased from a local supplier (Biotechno, Serang, Indonesia). Glacial acetic acid (CH_3_COOH), sodium hydroxide (NaOH), acetic anhydride (CH_3_CO)_2_O, ammonium phosphate (NH_4_)_3_PO_4_, and sulfuric acid (H_2_SO_4_) were purchased from Merck, Indonesia. WWAS was collected from the Arenga starch industry in Lebak Regency, Banten Province, Indonesia, and its characteristics are shown in Table 1.

### 2.2. Bacterial Cellulose Production

Fermentation for producing BC was conducted in static batch conditions. A total of 500 mL of WWAS as a culture medium was heated at 100 °C. Ammonium phosphate (NH_4_)_3_PO_4_ of 1% *w*/*v* and sucrose (food-grade white sugar) with concentrations of 100, 200, 300 and 400 g/L were added to the culture medium. Furthermore, glacial acetic acid was added to adjust the medium pH. Then, the culture medium was cooled at room temperature. After this, the inoculum of *Acetobacter xylinum* bacteria of 15% *v*/*v* was added to the culture medium and statically incubated at room temperature for 16 d. The resulting BC was collected, washed with distilled water, and immersed in 2% *w*/*v* NaOH solution for 60 min at a temperature of 80 °C to remove the bacterial cells. Furthermore, the BC was washed using distilled water, until the distilled water pH became neutral. Finally, the clean BC was dried at 105 °C until its weight was constant and the concentration (g/L) of BC was calculated using Equation (1).

(1) Production gL=BC weight gvolume of culture medium L

### 2.3. Cellulose Acetate Production

In this study, the CA synthesis process followed the procedures proposed by a previous study by Homem and Amorim [41] with several modifications. A mixture of 10 g of dry BC, 50 mL of glacial acetic acid, and 2 mL of sulfuric acid was stirred at 38 °C for 30 min. Then, as a solvent, acetic anhydride (AcAn) was added with the BC:AcAn ratios of 1:1, 1:2, 1:3, and 1:4 (*w*/*v*). The mixture was stirred again for 60 min at 38 °C. After the acetylation reaction, the solution was hydrolyzed by adding 4 mL and 8 mL of glacial acetic acid and distilled water, respectively. Then, the solution was reheated at 50 °C for 30 min under stirring throughout the process. Then, the solution was cooled at room temperature, centrifuged to separate the undissolved particles, and then filtered. The filtrate was transferred dropwise into excess distillate water and then the white solid CA was gradually formed. The resulting CA was filtered using a vacuum filter and washed with distilled water, until used distilled water pH became neutral. Finally, CA was dried at 60 °C in an oven under a vacuum overnight. The percentage of CA yield, the acetyl content, and the DS of CA were determined.

#### 2.3.1. Determination of the Percentage of CA Yield

The yield of CA is the ratio between the CA mass and the BC mass. Hence, the formula of the percentage of CA yield is shown in Equation (2).

(2) $$\mathrm{Yield}\;\left(\%\right)=\frac{\mathrm{CA}\;\mathrm{weight}\;\left(g\right)}{\;\mathrm{BC}\;\mathrm{weight}\;\left(g\right)}\times 100\%$$

#### 2.3.2. Determination of the Acetyl Content and the DS of CA 

The acetyl content and the DS of CA were determined through a method of alkaline saponification. In the method, 1.0 g of CA was dissolved in 40 mL of ethanol at 55 °C for 30 min. Then, 40 mL of 0.5 N NaOH solution was added. Furthermore, the mixture was heated again at 55 °C for 15 min and then kept at room temperature for 72 h. The excess alkaline in the solution was titrated against 0.5 N HCl, with phenolphthalein as an indicator. After 24 h, the small excess of acid was then back-titrated against 0.5 N NaOH until a pink color appeared. The acetyl content and DS were calculated using Equations (3) and (4), respectively [42].

(3) $$\%\;\mathrm{acetyl}=\frac{4.3\left[\left(A-B\right)N_{b}-\left(C-D\right)N_{a}\right]}{W}$$

(4) $$\mathrm{DS}=\frac{\left(3.86\times \;\%\;\mathrm{acetyl}\right)}{\left(102.4-\;\%\;\mathrm{acetyl}\right)}$$

In the equation above, A is the NaOH volume added to the sample; B is the NaOH volume added to the blank; C is the HCl volume added to the sample; D is the HCl volume added to the blank; N_a_ is the normality of HCl; N_b_ is the normality of NaOH; W is the sample weight; 4.3 is a factor used to calculate the % acetyl.

### 2.4. Product Characterizations 

#### 2.4.1. Fourier Transform Infrared (FT−IR) 

The functional groups and structures of BC and CA were analyzed using Fourier transform infrared (FT−IR) spectroscopy and a Nicolet iS5 spectrometer (Thermo Scientific, Waltham, MA, USA). The samples were prepared through the KBr disk method. Thirty-two scans at a resolution of 4 cm^−1^ on 400–4000 cm^−1^ regions were taken from each sample spectrum.

#### 2.4.2. ^1^H and ^13^C Nuclear Magnetic Resonance (^1^H NMR and ^13^C NMR)

Nuclear magnetic resonance (NMR) spectra were determined using ^1^H and ^13^C NMR spectroscopy (JEOL spectrometer that operated at 500 MHz, Peabody, MA, USA). Protons were determined using the solvent DMSO-d6 and chemical shifts were reported in parts per million.

#### 2.4.3. X-ray Diffractometry (XRD)

The crystallinity index of dry BC and CA powders was determined using XRD (Empyrean, Malvern Panalytical, Malvern, UK) at room temperature from 5 °C to 80 °C. The crystallinity index was determined using Equation (5) [43,44].

(5) $$\%\;\mathrm{Crystallinity}=\frac{\mathrm{Crystalline}\;\mathrm{area}\;\left(5-80^{{}^{\circ}}\right)}{\mathrm{Total}\;\mathrm{area}\;\left(5-80^{{}^{\circ}}\right)}$$

#### 2.4.4. Scanning Electron Microscope (SEM)

The characteristics of the BC and CA surfaces were analyzed using an SEM (JSM 6510LA-JEOL, Peabody, MA, USA), with an accelerating voltage of 10 kV. The SEM images of BC and CA were recorded at 20,000× magnification.

#### 2.4.5. Statistical Data Analysis

The experiments in this study were carried out in duplicate. The mean and standard deviations were calculated using Microsoft Excel software.

## 3. Results and Discussion

### 3.1. Production of BC in WWAS Medium

The fermentation of WWAS was carried out with variations in the sucrose additions and the initial medium pHs. The effects of the sucrose additions and the initial medium pHs on the thickness and weight of BC are shown in Table 2. 

The yield of BC under static conditions is affected by the carbon source concentrations [32]. In this study, sucrose was added as an extra carbon source. When sucrose was added in a low concentration, *Acetobacterium xylum* lacked the extra carbon source in the fermentation process, so the bacteria needed a long lag phase. On the other hand, if the sucrose was added in too high concentrations, the bacterial growth rate was inhibited [8]; consequently, the BC production rate was low. Based on Table 2, the production of BC by *Acetobacterium xylinum* was strongly affected by the sucrose addition. The optimal BC yield was obtained at a sucrose addition of 200 g/L and an initial pH of 4.5, with wet and dry weights of 505.6 and 43.6 g/L, respectively. At the same initial pH, the sucrose additions of 100, 300 and 400 g/L resulted in lower BC yields in terms of wet weights, which were 44, 166.4 and 21.2 g/L, respectively. Cakar et al. [12] investigated the effects of molasses concentrations (as a sucrose source) in a culture medium on bacterial cell growth and BC production with a strain of *Gluconacetobacter xylinus*. The study reported that an increase in molasses concentration to 200 mL/L at pH 3.0 increased the BC production to 0.572 g/L. However, a further increase in molasses concentration above 200 mL/L decreased the BC production. In addition, the molasses concentration of 300 mL/L resulted in no BC production. At very high sugar concentrations, unusual pathways of *G. xylinus* biomass production occurred [12]. Based on the information above, the BC yield in this study was higher than that in a study by Cakar et al. [12]. It might be due to the fact that this study used pure sucrose, while the study by Cakar et al., used molasse as a sucrose source.

Microorganisms require a specific pH level as an important factor to grow, so that they can produce a high yield of BC. The pH level in the static culture should be maintained below 6.0 [12]. Amor et al. [45] reported that a specific pH level can maintain a microorganism’s specific metabolic rate. Embuscado et al. [46] stated that a pH of 4.5 is the optimal pH for BC production, and a pH below 3.5 produces no BC. *Acetobacter xylinum* can grow in the range of pH levels of 3.5–7.5 with the optimal pH level of 4.3, while the cell metabolism of the bacteria will be disrupted under alkaline conditions [47]. Based on the contents of Table 2, besides sucrose additions, the production of BC was also affected by initial medium pHs (Table 2). The optimal initial pH was 4.5 when the sucrose addition value was 200 g/L. However, for the other values of sucrose additions, the optimal pH was not 4.5. For the sucrose additions of 300 g/L and 400 g/L, the best initial pH was 3.5, but at the initial pHs of 5.5 and 6.5, there was no BC production. For a sucrose addition of 100 g/L, the best initial medium pH was 4.5 with the BC yield of 44 g/L (wet weight), but it was much less than the BC yield obtained with a sucrose addition of 200 g/L and an initial pH of 4.5. Hence, the optimal initial pH for producing BC depended on the carbon source concentrations. Jagannath et al. [48] studied the effect of pH on BC production using coconut water as a fermentation medium. The study reported that fermentation at a pH of 4.0, a sucrose addition of 10% and an ammonium sulfate addition of 0.5% resulted in the maximum BC production with a thickness of 10.2 ± 0.26 mm. On the other hand, at a pH of 3.5, after incubation for 20 days, the resulting BC demonstrated a very thin thickness (~1 mm). Furthermore, when glucose and mannitol were used as extra carbon sources, the optimal pHs were 5.5 and 6.5, respectively [49].

**Figure 1:**
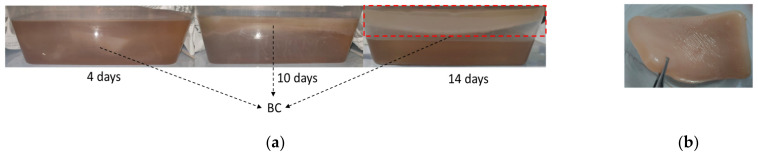

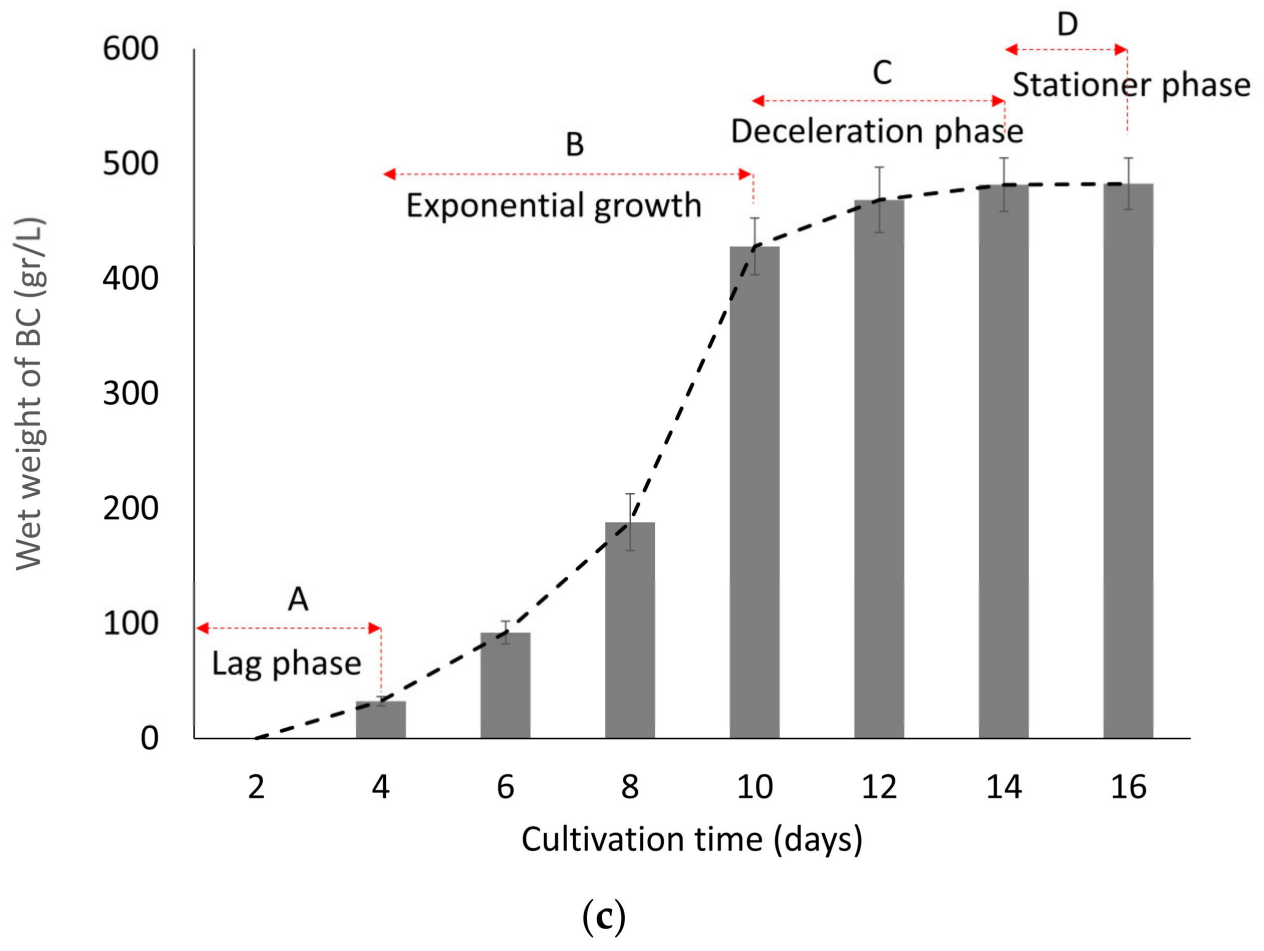
(**a**) The growth of BC during incubation, (**b**) the BC product after purification; (**c**) the wet weight of BC with a sucrose addition of 200 g/L and an initial medium pH of 4.5.

From Table 2, it can be observed that the maximum amount of BC was produced following fermentation with the sucrose addition of 200 g/L and the initial medium pH of 4.5, with a cultivation time of 14 days. The resulting BC had a thickness of 21 mm and a wet weight of 505.6 g/L of culture medium. The growth of BC during incubation under these conditions is shown in Figure 1.

During incubation, the BC morphology transformed from a floccus to a membrane. The microfibers intertwined and aggregated with each other to form irregular meshes or flocculent structures. The formation of the BC membrane can be observed on the surface of the medium. It means that the BC production was directly related to the surface area of the air–liquid interface, which is shown in Figure 1a. A longer incubation time resulted in a thicker BC membrane. The sub-fibrils of cellulose, which were continuously released from linearly ordered pores at the surface of the *Acetobacter xylinum*, crystallized to form microfibrils. Therefore, the BC pellicle that supported the population of *Acetobacter xylinum* overlapped and intertwined to form parallel, but disorganized, planes of cellulose ribbons [50]. Furthermore, BC assembled into a thick membrane on the surface of the medium. The freshly harvested BC was primrose yellow, as shown in Figure 1b. During the static cultivation process, BC growth can be observed in four phases, as shown in Figure 1c. At the first 4 days of incubation, as shown in zone A, the BC films slowly formed in the lag phase. This was because the *Acetobacter xylinum* bacteria were still adapting to the utilization of the substrate, so the growth rate of the *Acetobacter xylinum* bacteria was low. The majority of BC was produced in the exponential phases (in zone B). In zone B, the bacteria strains grew exponentially because the culture medium still contained sufficient nutrients so that more BC films were formed. When the BC films became thicker, which reduced the oxygen supply to the strain, and the nutrients (carbon sources) in the medium decreased, the bacterial activity was disturbed, so BC formation decreased (in zone C). The formation of BC finished when the pellicle grew downward and entrapped all the bacteria, so the bacteria became inactive because there was no oxygen supply [51] (in zone D). The BC formation process with static conditions was controlled by air supply from the surface of the medium and the yield of BC was moderately affected by the concentration of the carbon source [32]. The longer the incubation time, the more BC was formed, along with the formation of hydrogen and C-H bonding [52].

### 3.2. Cellulose Acetate Production

The acetylation of BC, which was derived from WWAS, was carried out with various BC:AcAn ratios. In Table 3, it can be observed that the difference in the BC:AcAn ratios resulted in the different values of the yield, acetyl content, and DS of CA.

Based on Table 3, a decrease in the BC:AcAn ratio from 1:1 to 1:3 successfully increased the CA yield from 20.46 to 81.49%. AcAn is an acetylating agent, so an increase in AcAn concentration can improve the acetylation of cellulose; thus, an increase in the yield of CA was obtained [53]. AcAn triggered the displacement of hydroxyl groups to acetyl groups, so the acetyl content increased [54]. In addition, an increase in the BC:AcAn ratio from 1:1 to 1:4 increased the acetyl content and degree of substitution (Table 3). The acetyl content represents the amount of esterified acetate in the cellulose chain, which determines the value of the degree of substitution. The degree of substitution represents the average number of hydrogen atoms in the hydroxyl group (−OH), which are transformed to acetyl groups in each anhydroglucose residue [39]. However, a BC:AcAn ratio of 1:4 resulted in a lower CA yield than the ratio of 1:3. This might have been caused by the excess of acid content in the solution, which caused the redissolving of the CA precipitates [55].

The DS value indicates the ease of the component to be degraded. A low DS value indicates that the component is more easily degraded [42], so by controlling the DS value, the biodegradation rate and solubility of CA can be modified [56]. CA with a DS ≈ 3 does not decompose naturally, while that with a DS ≈ 2.5 slowly decomposes, and that with a DS < 2.2 readily decomposes [57]. Thus, this study showed that the resulting CA was soluble in acetone and chloroform, but insoluble in water. In line with this study, a study by Battisti et al. [58] also reported that CA samples with a DS between 2.08 and 2.82 showed good solubility either in acetone or chloroform at an ambient temperature (25 °C), while the CA samples with a DS between 2.2 and 2.7 were soluble in acetone. DS values between 2.2 and 2.7 and above 2.8 belonged to cellulose diacetate and triacetate, respectively. Based on this information, the CA produced in this study belonged to cellulose diacetate. According to the Indonesian national standard, CA ideally contains an acetyl content of 39–40% and is soluble in organic solvents [39]. From Table 3, it can be observed that this type of CA was obtained by the acetylation process at a BC:AcAn ratio of 1:3, due to its acetyl content of 39.82%.

### 3.3. Product Characterization

#### 3.3.1. FT–IR Spectroscopic Analysis

**Figure 2:**
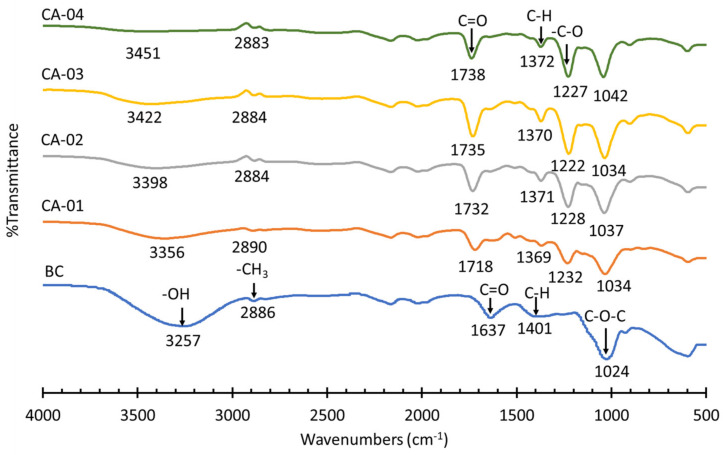
FT–IR spectrum of BC from WWAS and CA derived from the acetylation of BC.

The functional groups of BC from WWAS and CA derived from the acetylation of BC were measured using FT−IR, as shown in Figure 2.

As displayed in Figure 2, the absorption peaks at 3257, 2886, 1637, 1401, and 1024 cm^−1^ were attributed to the stretching vibration of hydroxyl groups (–OH), the C–H stretching vibrations of methyl groups (–CH_3_), carboxylic acids (C=O), C–H bending vibrations, and C–O–C ester bonds, respectively [12,59]. As can be observed in Figure 2, the spectra for the acetylated samples showed a set of peaks that confirmed a modification in the BC structure. In this study, the spectra for CA-01, CA-02, CA-03, and CA-04 were very similar. Confirmation of the successful acetylation process of BC to CA was identified by the appearance of new peaks in the CA samples, namely 1718–1738 cm^−1^, 1369–1372 cm^−1,^ and 1222–1232 cm^−1^, which were related to the C=O stretching of carbonyl esters, the C–H bond in O−(C=O)−CH_3_ and the −C−O stretching of acetyl groups, respectively. In addition, another important aspect observed in the CA spectrum was a decrease in the intensity of O–H stretching at band 3400–3200 cm^−1^, when compared to BC [54,56,58,60]. The decrease in the intensity of hydroxyl stretching occurred because the hydroxyl groups were replaced by the acetate groups during the acetylation reaction [56,58]. The CA with a higher BC:AcAn ratio in the acetylation process resulted in a lower intensity of the O–H stretching characteristic. The decrease in the intensity of the hydroxyl band gradually increased the reaction yield, depending on the degree of substitution (DS). Therefore, there was a direct relationship between the DS value and the yield value, as shown in Table 3. In this study, the yield and DS of CA ranged from 20.46 to 81.49% and 1.444 to 2.456, respectively. The absence of absorption peaks between 1840 and 1760 cm^−1^ indicated the absence of unreacted acetic anhydride. The absence of absorption peaks around 1700 cm^−1^ showed that the products were also free of acetic acid [54,58,61].

#### 3.3.2. NMR Analysis

**Figure 3:**
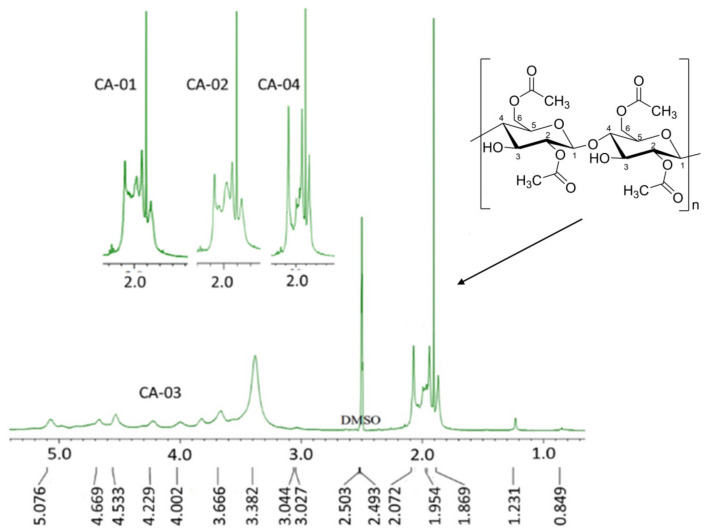
^1^H NMR spectra of CA samples.

The acetylation of BC was confirmed using the ^1^H dan ^13^C NMR spectrum of the cellulose acetate products. The ^1^HNMR complete spectra of CA-03 and carbonyl carbon of CA-01, CA-02, and CA-04 are shown in Figure 3. As observed in the spectrum of CA-03, the appearance of peaks in the region of 2.1–1.8 ppm that correspond to the methyl groups (CH_3_) of the acetyl groups indicated that the acetylation of BC was successfully achieved [54,60]. The signals at 2.072, 1.954, and 1.906 ppm were related to the carbonyl carbon at C6, C2, and C3, respectively. The O–acetyl group signal, which can be clearly observed, provided us with the possibility to predict the three hydroxyl groups’ reactivity. From the ^1^HNMR spectrum, it can be observed that the calculated integration of carbonyl carbon from the acetyl moieties, which replaced the three OH groups, showed the different reactivities from the three hydroxyl groups at C2, C3, and C6. Thus, the order of reactivity can be predicted as follows: C3–OH > C2–OH > C6–OH. All the CA samples (CA-01, CA-02, CA-03, and CA-04) showed similar peak spectra. The peaks in the region of 3.5–5.1 ppm corresponded to the seven protons in the anhydroglucose ring of cellulose. The peaks around 2.50–2.49 and 3.382 were assigned to the protons of DMSO and residual water, respectively.

**Figure 4:**
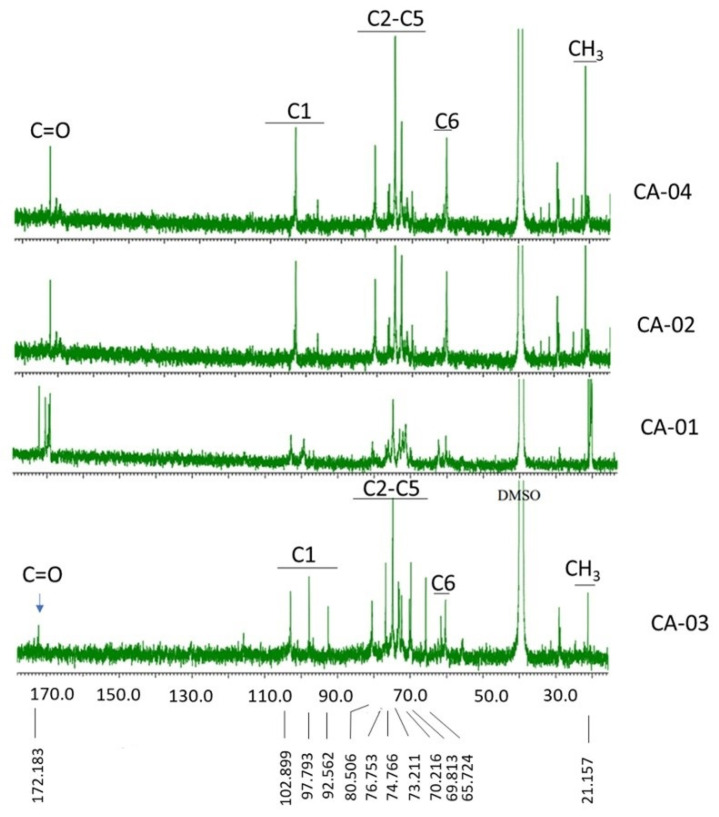
^13^C NMR spectra of CA.

Figure 4 shows the ^13^CNMR spectra for the acetylated samples (CA-01, CA-02, CA-03, and CA-04). In the spectrum of CA-03, the two signals that appeared at 172.183 ppm and 21.157 ppm were associated with carbonyl (C=O) and methyl (CH_3_) carbons in acetate groups, respectively, which resulted from acetylation reactions. In the same spectrum, the C1 carbon signals appeared at 92.562, 97.793, and 102.899 ppm, and the C6 carbon signals appeared at 60.320, 61.558, and 65.724 ppm. However, the C1–C5 carbon signals appeared at 70–80 ppm. The spectra of C=O, C1, C2–C5, C6, and C–Me are shown in Table 4.

#### 3.3.3. X-ray Diffraction (XRD) Analysis

**Figure 5:**
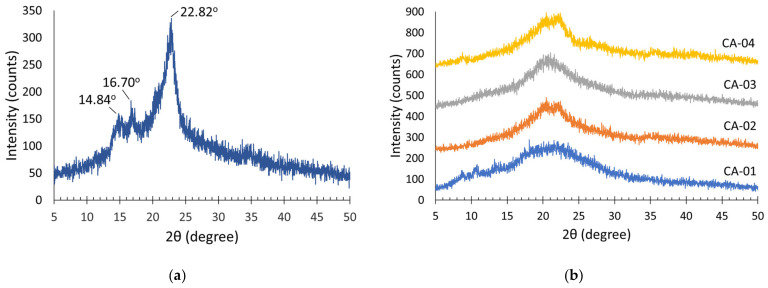
XRD patterns of (**a**) BC; (**b**) CA.

The crystallinity of the BC structure was analyzed using XRD. The XRD patterns of BC isolated from WWAS and acetylated BC are shown in Figure 5a,b. From Figure 5a, it can be observed that the diffraction patterns of BC synthesized using WWAS as a medium showed three main diffraction peaks at 2θ angles of 14.84°, 16.70°, and 22.82°. These signals were attributed to the crystallographic planes, which were represented as Miller indices of 100, 010, and 200. These indicated the type of cellulose I_α_; this was the indication of bacterial cellulose [9]. Similar results were reported by some previous studies [9,62,63]. The crystallinity index of BC in this study was around 79.6%.

The acetylation of BC caused significant changes in the arrangement of the cellulose chains, as shown in Figure 5b. The XRD pattern of CA-01 showed diffraction peaks at 2θ angles of 9°, 11.36°, and 18.8°. CA-02 showed weak diffraction peaks at 8°, 18°, 20°, and 22°, while CA-03 and CA-04 showed weak diffraction peaks at 9°, 18°, 20°, and 22°. Two weak diffraction peaks at 9° and 18° are characteristic peaks for cellulose diacetate [53,64]. The appearance of the main peak was located at 8°, due to the replacement of the hydroxyl groups by acetyl when cellulose was acetylated and was related to an increase in the interfibrillar distance and also a breakdown of the microfibrillar structures. This peak represented the main characteristics of the semicrystalline structure of acetate [56,58].

The acetylation process occurs simultaneously with the process of eroding the BC crystal structure. Therefore, the degree of crystallinity after the acetylation process should be lower compared with the original BC. The higher the amount of acetic anhydride added, the lower the degree of CA crystallinity. The acetylation process begins in the amorphous region of BC, and then spreads outwards toward the center of the cellulose crystal. As the reaction progresses, swelling of several acetate chains occurs in the cellulose fiber, causing it to become a solid, and finally, a CA crystal structure is formed [65]. The degree of crystallinity for CA-01, CA-02, CA-03, and CA-04 was 47.8%, 39.4%, 36.7%, and 34.2%, respectively. The low and wide peaks showed that CA had a low degree of crystallinity.

#### 3.3.4. Scanning Electron Microscope (SEM)

**Figure 6:**
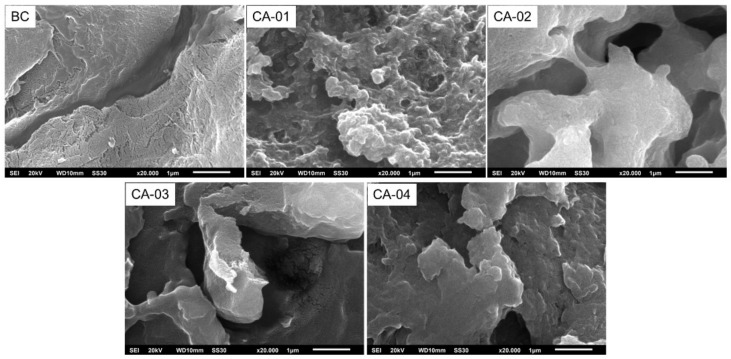
SEM micrographs of BC and CA.

Figure 6 shows the SEM image of the BC produced from WWAS and CA that resulted from the acetylation of BC, respectively. The surface morphology analysis showed a significant difference between BC and CA. BC in the dry state had a tough crystalline structure, which was dense, nonporous, and uniformly distributed. This was similar to the BC morphology reported by Du et al. [59]. Figure 6 shows that the acetylation process caused substantial morphological changes in the microstructure of the CA samples. During the acetylation and hydrolysis reactions, the cellulose fibers underwent swelling and formed a sponge structure. As the amount of acetic anhydride added in the acetylation process increased (CA-04 > CA-03 > CA-02 > CA-01), the morphology of the CA fibers became more irregular, more holes were formed and almost all of the cellulose structure was destroyed. The morphology of the CA-01 fibers seemed to be homogeneous, with a few holes formed that were small in size. This indicated that the acetylation reaction mainly occurred only on the surface of cellulose. The morphology of CA-02, CA-03, and CA-04 showed that more holes were formed and penetrated the entire particle, so their sponge structures were very clearly visible. This indicated that the acetylation reaction simultaneously occurred in the interior and on the surface of the cellulose fibers.

## 4. Conclusions

Wastewater of the Arenga starch industry (WWAS) was successfully reused for the synthesis of BC under static incubation by *Acetobacter xylinum*, with a sucrose addition of 200 g/L, an initial medium pH of 4.5, and a cultivation time of 14 d. The resulting BC had a thickness of 21 mm and a wet weight of 505.6 g/L of culture medium. The BC was converted into CA with varying BC:AcAn ratios. The higher the amount of AcAn added to the acetylation process, the higher the yield, the acetyl content, and the DS of CA. In the acetylation process, the optimal BC:AcAn ratio was 1:3, resulting in the CA product with a yield of 81.49%, an acetyl content of 39.82%, a degree of substitution (DS) of 2.456, and a degree of crystallinity of 36.7%. The FT−IR analysis showed the successful acetylation process of BC to CA, which was identified by the new peaks in the CA samples at 1718–1738 cm^−1^, 1369–1372 cm^−1,^ and 1222–1232 cm^−1^. The spectrum of ^1^HNMR showed characteristic peaks for the methyl group (CH_3_) of the acetyl groups in the region of 2.1–1.8 ppm. The SEM analysis showed that as the amount of AcAn added in the acetylation process increased, the morphology of the CA fibers became more irregular, more holes were formed and almost all of the cellulose structure was destroyed. The results of the FT–IR, NMR, SEM and XRD analysis showed that the acetylation process of BC, which resulted from WWAS, to CA was successfully achieved. Therefore, the results of this study confirmed the hypothesis in which WWAS has the potential as a fermentation medium for producing inexpensive and environmentally friendly biomaterials (BC and CA) that can be developed on a large scale.

## Figures and Tables

**Table 1:** WWAS characteristics.

Parameters	Methods	Values *
pH (25 °C)	A digital pH meter	6.9
Dissolved oxygen (DO)	SNI 06-6989.14-2004	0.73
Total dissolved solid (TDS)	A digital TDS meter	258
Total suspended solid (TSS)	Gravimetric analysis	2750
Biology oxygen demand (BOD)	SNI 6989.72-2009	3050
Chemical oxygen demand (COD)	SNI 6989.2-2009	6394
Ammonia	IK 7.2.1.2 KA (spectrophotometric)	7.2

* Note: The unit of all the parameter values is mg/L, except for the unit of the pH parameter value.

**Table 2:** Effect of the sucrose addition and initial medium pH on BC.

Sucrose Addition (g/L)	Initial Medium pH	BC		
		Thickness (mm)	Wet Weight (g/L)	Dry Weight (g/L)
	3.5	2	22	0.16
100	4.5	5	44	2.2
	5.5	1.5	16	0.016
	6.5	-	-	-
	3.5	16	320.6	28.4
200	4.5	21	505.6	43.6
	5.5	8	150.2	5.4
	6.5	1	7.6	0.002
	3.5	12	181.2	11.6
300	4.5	10	166.4	8.8
	5.5	-	-	-
	6.5	-	-	-
	3.5	3.0	28	0.32
400	4.5	2.5	21.2	0.20
	5.5	-	-	-
	6.5	-	-	-

**Table 3:** Percentage of yield, acetyl content, and DS of CA.

Sample	The Ratio of BC:AcAn (*w*/*v*)	Yield of CA (%)	Acetyl Content of CA (%)	Degree of Substitution (DS) of CA	Solubility		
					Water	Acetone	Chloroform
CA-01	1:1	20.46	27.87	1.444	-	+	+
CA-02	1:2	52.82	35.44	2.043	-	+	+
CA-03	1:3	81.49	39.82	2.456	-	+	+
CA-04	1:4	74.13	41.65	2.645	-	+	+

**Table 4:** Solid-state ^13^C chemical shifts of the acetylated samples.

Sample	C=O	C1	C2–C5		C6	C–Me
CA-01	169.092	99.357	71.281	72.126	60.291	20.130
	169.418	102.880	72.990	74.833	62.297	20.245
	170.407					20.339
	172.125					20.639
						21.119
CA-02	172.173	96.689	73.009	74.833	60.300	20.283
		102.899	76.513	76.849		20.667
			80.486			21.147
						22.184
						24.555
CA-03	172.183	92.562	69.813	70.216	60.320	21.157
		97.793	72.366	73.009	61.558	
		102.899	73.211	74.766	65.724	
			76.753	80.506		
CA-04	172.163	96.689	74.766	74.862	60.310	21.138
		97.793			61.087	22.174
						24.545

## Data Availability

Data available upon request.
